# Uncovering mnestic problems in help-seeking individuals reporting subjective cognitive complaints

**DOI:** 10.1038/s41598-023-42527-x

**Published:** 2023-09-14

**Authors:** M. Werrmann, A. Schegolevska, M. Eid, M. Niedeggen

**Affiliations:** 1 Division of Experimental Psychology and Neuropsychology, Department of Educational Science and Psychology, Freie Universität Berlin, Habelschwerdter Allee 45, 14195 Berlin, Germany; 2 Division of Methods and Evaluation, Department of Educational Science and Psychology, Freie Universität Berlin, Berlin, Germany

**Keywords:** Psychology, Health care

## Abstract

In individuals with subjective cognitive impairments (SCI) the risk for the development of a neurodegenerative disease is assumed to be increased. However, it is not clear which factors contribute to the expression of SCI: Is it related to the cognitive resources already challenged, or is the psycho-affective state of more relevance? Using a novel online assessment combining self-report questionnaires and neuropsychological psychometric tests, significant predictors for the level of complaints were identified in two samples of elderly individuals: Help-seekers (HS, n = 48) consulting a memory clinic and a matched sample of non-help-seekers (nHS, n = 48). Based on the results of the online assessment, the SCI level was found to be significantly determined by the psycho-affective state (depressive mood) in the nHS group, whereas cognitive performance (cued recall) was the main predictor in the HS group. The predictive value of recall performance, however, is more-strongly expressed in memory tests which reduce the impact of compensatory strategies (face–name-association vs. word lists). Our results indicate that the problem-focused behavior of help-seeking individuals is also associated with a higher sensitivity for cognitive deficits—which can be uncovered with an appropriate psychometric test. Considering these factors, the conversion risk in individuals with SCI can probably be determined more reliably.

## Introduction

Subjective cognitive impairment (SCI) describes the presence of subjective cognitive deficits which cannot be related to an acute event. The complaints are strictly based on the perspective of the individual, and are not associated with significant deficits in psychometric tests^1,2^. In an aging society, SCI is increasingly prevalent. About one quarter of objectively unimpaired adults above the age of 60 suffer from SCI^2^. A recent meta-analysis of prospective studies found that individuals with SCI have an about two-fold increased risk of developing actual cognitive impairment or dementia compared to participants without SCI^3^. The dissociation between the subjective und objective performance has been attributed to compensatory processes: According to Jessen and colleagues^1^ individuals with SCI notice changes in the ease of using cognitive functions and performance in daily life. Due to the successful application of more-demanding cognitive strategies, however, the performance of these individuals is still in an age-related normal range^4^.

Despite of its clinical relevance, the use of a clinical concept of SCI has also been questioned. The first problem refers to the reliable assessment of cognitive impairments in the diagnostic process^5^. SCI has often been conceptualized as subjective memory complaints and measured in the form of single item questions targeting perceived memory problems specifically^6,7^. However, a reliable measurement of SCI has to incorporate a range of cognitive domains which are adequately assessed with multiple questions related to daily activities^8^. If questions are primarily related to mnestic problems, neurodegenerative processes not linked to an Alzheimer’s dementia will not be addressed^9^. Recently developed questionnaires consider these requirements and include different cognitive domains, such as attention or executive functions^10–12^. This study takes advantage of the CPI (Complainer Profile Identification^13^) which shares the psychometric qualities of established questionnaires^14^ and has already been related to the performance in attention tasks^15^.

A second, more serious problem is related to the validity of SCI measurements. Although individuals with SCI do not necessarily fall below clinical cut-off values of psychometric tests, the subjective problems in daily functions^16^ and the expression of SCI should be (and have been) related to the level of test performance^17,18^. A corresponding meta-analysis indicates a rather modest linkage between subjective and objective performance^19^ which is probably due to the necessity of using compensatory strategies in psychometric tests. A more reliable association has been identified between the level of cognitive complaints and the psycho-affective state. In particular, symptoms of depression^20^ and self-focused attention^21^ served as important predictors for the level of complaints. Recent studies suggest that depressive mood, even if it does not reach the level of a clinical depression, can impose a negative bias on the perception of one’s cognitive capabilities^13,22^. Consequently, the influence of these variables has to be considered when conclusions about cognitive impairments were made based on SCI measurements.

Finally, one has to consider that the interplay between cognitive performance, depressive mood, and subjective impairments might be expressed differently in different population groups. Relevant moderator variables, for example, are the level of health concern and precautionary behavior: In help-seeking individuals (HS) which visited a memory clinic, performance in attention tests was found to be associated with the SCI level, whereas a corresponding relation was not found in a matched sample of non-help-seekers^15,22^. Importantly, these studies also suggest that problem-focused behavior in help-seekers^23^ is frequently associated with a higher level of depressive mood. The latter might contribute to cognitive problems, such as divided attention problems^15^.

In sum, the identification of the factors which determine the level of subjective cognitive complaints in individuals remains controversial. Considering that different factors affect the expression of SCI, Jessen and colleagues^24^ recently proposed a differentiation of SCI subtypes (reversible, stable, or progressive course) which differ regarding the risk for developing a neurodegenerative disease. The proposed classification will depend on several factors, such as the aforementioned ‘profile’ of cognitive complaints, temporal characteristics, or help-seeking behavior.

In this study, we aim to identify cognitive and psycho-affective predictors for the expression of SCI in different populations, help-seekers and non-help-seekers. Moreover, we ask whether the choice of the psychometric instrument measuring the cognitive state determines the predictive value. As mentioned above, it has been proposed that individuals with SCI apply compensatory strategies in daily living—and this might also apply to the neuropsychological assessment based in psychometric tests^4^. This motivates the question whether the relationship between the level of cognitive complaints and performance will be modulated if compensatory strategies cannot be applied successfully. As far as we know, this issue has not been addressed in previous studies. To this end, we developed an online screening tool for neuropsychological functions (“Neuropsychological Online Screening”, NOS). The sensitivity of self-administered tools has recently been highlighted^25^, and identified as a promising approach in monitoring age-related changes.

**Figure 1:**
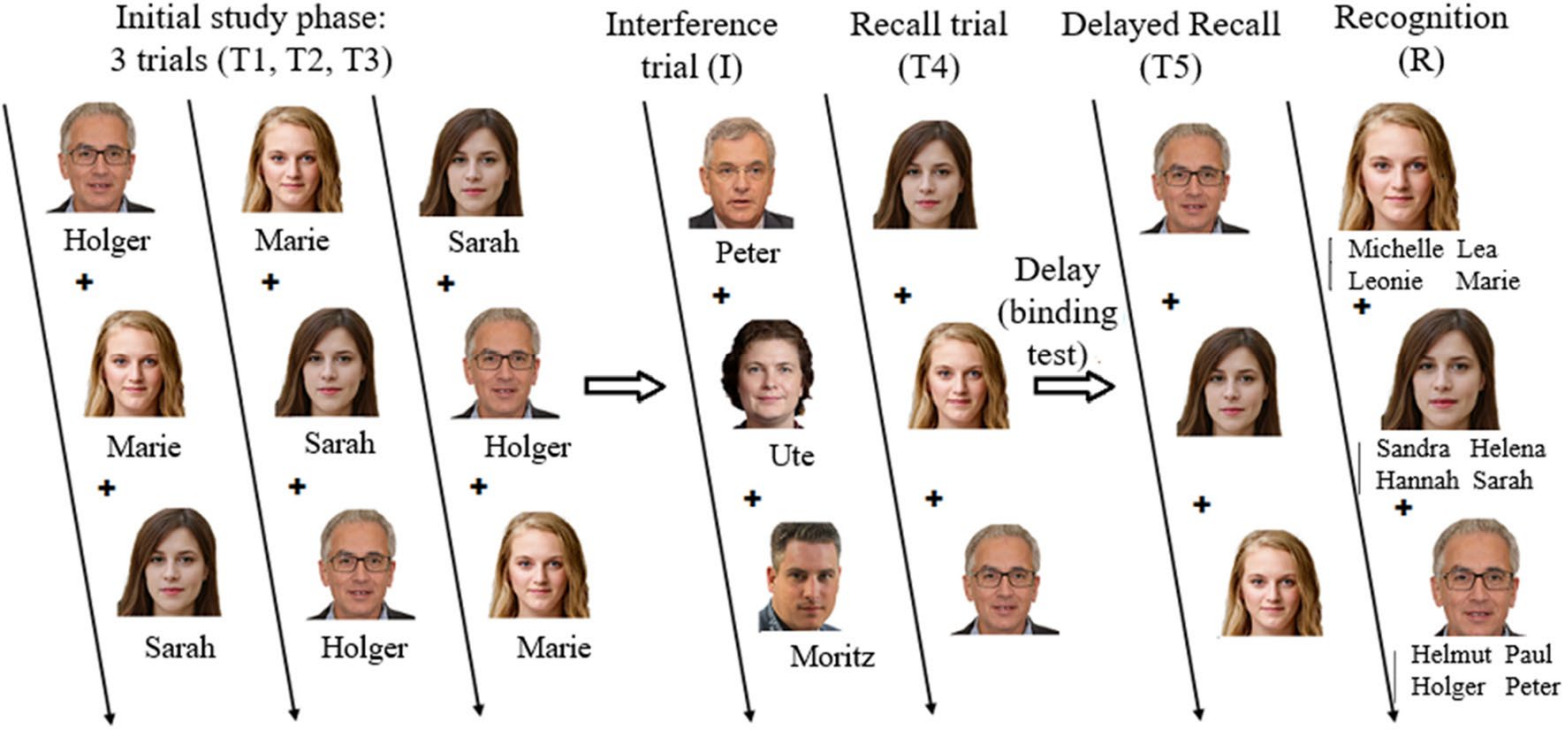
FNAT procedure: the different phases of the FNAT. FNAT (Face-Name Association Test). In the initial study phase, a portrait (face) and a corresponding name are presented. In the following test phase, the face is presented and the initial letter of the corresponding name must be entered. This procedure is repeated three times (T1, T2, T3). In a single interference trial, different face–name associations are learned. In the recall trials, the faces from the initial study phase are presented—either immediately following the interference trial (T4) or after a delay of approximately 15–20 min (T5). For the recognition trial, faces from the first learning list are presented and the corresponding name has to be selected out of four choices. All faces are computer-generated.

The NOS combines the registration of subjective problems via CPI and two tests for mnestic performance. Beside of a visual working memory test, a face–name-association test (FNAT) was implemented (see Fig. 1) which shares the characteristics of the established verbal learning-and-memory tests: Following repetitive presentation of the stimuli defining the learning curve, free-recall ability and recognition performance was measured. These parameters are known to be clinically relevant indicators for mnestic deficits^26^. In contrast to verbal learning-and memory tests, a face–name-association task has a higher ecological validity since retrieving these associations is a common task in daily living^27^. Additionally, we suppose that the mnestic performance in individuals reporting SCI can be estimated more reliably: In contrast to verbal tasks, the utilization of compensatory memory strategies is less possible^28^. This difference primarily relies on the random association between the complex visual stimulus (face) and a personal name.

The performance in the online screening was registered in a matched sample of individuals visiting a neuropsychological consultation hour (HS) and controls (nHS). For help-seekers, results of a thorough neuropsychological examination were additionally available.

Our research questions are the following:

1. Can we replicate previous findings indicating that the predictive value of cognitive performance on the level of SCI is restricted to the help-seeking individuals? In concordance with previous studies^15^, we expect that the concerns in help-seekers (HS) resulting in a problem-focused behavior will increase the sensitivity for cognitive limitations. Since previous studies suggest that the relation between objective test performance and subjective cognitive problems is driven by the level of depressive mood, a corresponding mediator effect is expected^15^. For the group of non-help-seekers (nHS), the cognitive performance level is not assumed to be related to the level of subjective limitations.
2. Does the predictive value of objective test performance—hypothesized for help-seeking individuals—depend on the choice of the psychometric instrument? Recall and recognition performance are sensitive indicators for several neurodegenerative processes, and verbal learning-and-memory tests usually provide the gold-standard in neuropsychological diagnostics. However, these tests did not serve as predictors for the level of SCI in previous studies^13,15,18^. We hypothesize that psychometric tests that are less prone for compensatory processes, for instance a face–name association test, are more closely related to the level of SCI.

## Results

Table 1 summarizes the characteristics of the two groups, HS (n = 48) and nHS (n = 48). The results of the pairwise comparison provide information on systematic differences in different domains.

**Table 1:** Descriptive statistics for HS and nHS and inference tests for group differences. *CPI* Complainer Profile Identification, *GDS* Geriatric Depression Scale, *FNAT* NOS face-name association test recall; ^1^Phi-coefficient; ^2^Cramer v; ^3^Cohen’s d; Gender: The number of females and males was comparable in the HS-group (26 female, 22 male) and the nHS-group (24 female, 24 male).; Education: The distribution of education levels was comparable between the HS-group (< 10 years: 6, 10 years: 14, > 10 years: 28) and the nHS-group (< 10 years: 8, 10 years: 16, > 10 years: 24); significance level: *p < 0.05, **p < 0.01, ***p < 0.001.

	HS		nHS		p	Effect size	95% Confidence interval	
	Mean	SD	Mean	SD			Upper limit	Lower limit
Gender	See note		See note		0.683	− 0.042^1^		
Education	See note		See note		0.695	0.087^2^		
Age (in years)	50.63	14.16	52.77	15.29	0.477	0.146^3^	− 0.255	0.546
CPI	3.24	0.73	2.27	0.48	< 0.001***	− 1.573^3^	− 2.028	− 1.111
GDS	7.85	3.98	2.62	2.86	< 0.001***	− 1.507^3^	− 1.958	− 1.050
FNAT								
Trial 1–3	19.56	8.01	22.71	7.56	0.051	0.404^3^	− 0.001	0.807
Interference	4.00	2.68	5.48	2.73	0.009**	0.547^3^	0.138	0.953
Recall	6.71	3.19	8.17	3.19	0.027*	0.458^3^	0.051	0.862
Delayed recall	7.06	3.35	8.19	3.17	0.094	0.345^3^	− 0.059	0.747
Recognition	9.79	2.54	10.48	1.95	0.139	0.304^3^	− 0.099	0.706

As for the matching factors “age”, “gender”, and “education”, statistics confirmed that groups were comparable. The help-seeking behavior, however, was reflected in the self-reports and affective state: HS showed a higher level of subjective complaints in the CPI score, and the depressive mood as measured with a short version of the Geriatric Depression Scale (GDS)^29^ was enhanced. In the HS-group, 20 participants exceeded a critical score of eight points (out of a maximum of 15 points) signaling a moderate depression^30^, in the nHS-group only three individuals exceeded the critical score.

In the psychometric testing, the comparison of the level of test performance was focused on FNAT scores of interest: Descriptively, the individuals in the nHS group showed a better learning rate (FNAT sum: trial 1–3) and were less affected by proactive interference (FNAT interference) as compared to the HS group. This advantage also applies for the recall (FNAT recall, FNAT delayed recall) and recognition (FNAT recognition) performance. According to the effect sizes, the consistent group differences were mostly expressed for the interference and recall effect. The latter value (FNAT recall) was also identified as the most sensitive mnestic test score for the subsequent regression analysis (see methods).

In a subgroup of HS individuals (n = 35), the recall performance as estimated by the FNAT was expanded by the corresponding test value as estimated by standardized memory tests (either VLMT^31^ or CERAD^32^). In both tests, the evaluation of individuals’ test scores includes a transformation to a z-score considering the factor ‘age’. The mean z-score obtained in this subgroup (*M* = − 0.06, *SD* = 1.04) indicated that the overall recall performance is comparable to the normative group. The addition of these scores also allow an estimation of the convergent validity of the FNAT and the standardized verbal learning-and-memory test. The partial correlation considered the factor ‘age’ which is not considered in the FNAT score: The resulting correlation coefficient between the scores in the tests—controlling for age—was *r* = 0.42 (*p* = 0.014). Note that the necessity for controlling the factor ‘age’ was confirmed by its relevant correlation with the FNAT recall score (*r* = − 0.29, 95% confidence interval = [− 0.47, − 0.10]).

### Predicting the level of subjective cognitive complaints

In the first step of analysis, variables which serve as predictors for the level of subjective impairment were identified by running a linear regression. Given the sample size, the number of predictors tested was reduced to five. Following our hypothesis, the analysis included depressive mood (GDS score), recall performance in the FNAT (FNAT recall), and the factor “group” (HS vs. nHS) variable. In order to account for the differences between HS and nHS, interactions with the group factor were considered in the regression. Finally, the factor ‘age’ was included as control variable.

**Figure 2:**
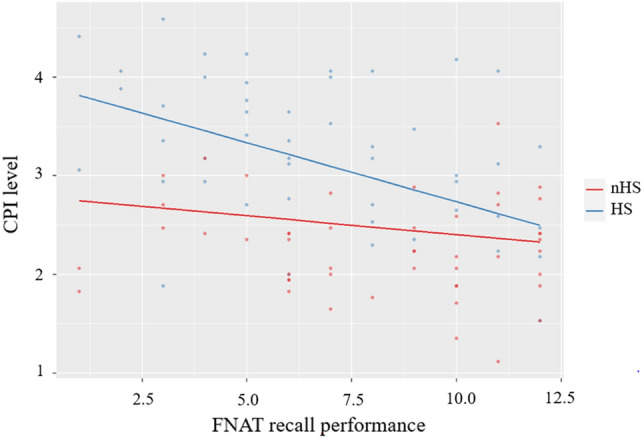
CPI level as a function of the FNAT recall performance. The functions differ between the groups defined by different help-seeking behavior. *CPI* Complainer Profile Identification, *FNAT* face–name association test. There is a greater relation between CPI level and FNAT recall performance in the HS-group than the nHS-group.

The result of the first regression model (see Table 2) shows that the R^2^ value (*R*^*2*^ = 0.755, *R*^*2*^_*corr*_ = 0.570) is significantly different from 0 (*F* (6,89) = 19.70; *p* < 0.001). All independent variables together explain 57% of the variance of CPI, which is a significant increase as contrasted to a regression model only considering the factor ‘age’ (*R*^*2*^ = 0.036, *R*^*2*^_*corr*_ = 0.026, Δ*R*^*2*^ = 0.534, p < 0.001). In line with the descriptive statistics, the significant factor ‘group’ shows a higher CPI level in the HS group. Moreover, higher depressive mood (GDS) and younger age (age) correspond with a higher CPI level. Most importantly, the impact of the performance in the memory test (FNAT recall) depends on the group assignment (significant interaction effect): A plot of the interaction in the regression model (see Fig. 2) supports the notion that cognitive performance is related to the subjective impairment exclusively in the HS group. There is no significant interaction between depression and group.

**Table 2:** Regression analysis for overall level of SCI (CPI). *CPI* Complainer Profile Identification, *GDS* Geriatric Depression Scale, *FNAT* NOS face-name association test recall; significance level: *p < 0.05, **p < 0.01, ***p < 0.001

	B	SE of B	β	p
Constant	2.953	0.343		< 0.001***
Group	1.331	0.313	0.852	< 0.001***
Age	− 0.011	0.004	− 0.199	0.009**
GDS	0.073	0.028	0.405	0.009**
FNAT recall	− 0.038	0.025	− 0.158	0.130
Group × FNAT recall	− 0.082	0.035	− 0.421	0.021*
Group × GDS	− 0.035	0.034	− 0.214	0.306

R^2^ = 0.755; R^2^_corr_ = 0.541; *p* < 0.001

The significant interaction effect between group and FNAT recall performance justifies a separate regression analysis for each group.

In the model for the group HS (Table 3), the aforementioned variables explained 30.4% of the total CPI variance (R^2^ = 0.348, *R*^*2*^_*corr*_ = 0.304, *F* (3,44) = 7.84, *p* < 0.001). Again, this result reflects a significant increase as contrasted to a regression model only considering the factor ‘age’ (*R*^*2*^ = 0.038, *R*^*2*^_*corr*_ = 0.017, Δ*R*^*2*^ = 0.310, p < 0.001). Within this group, two significant independent variables were identified: The level of SCI decreased with the age of the participants (variable age), and was increased if the recall performance was reduced (variable FNAT recall). The effect of depressive mood was not significant.

**Table 3:** Regression analysis for level of SCI in HS. *GDS* Geriatric Depression Scale, *FNAT recall* NOS face-name association test recall; significance level: *p < 0.05, **p < 0.01, ***p < 0.001

	B	SE of B	β	p
Constant	4.806	0.508		< 0.001***
Age	− 0.019	0.007	− 0.364	0.008**
GDS	0.034	0.023	0.188	0.137
FNAT recall	− 0.132	0.030	− 0.577	< 0.001***

R^2^ = 0.348; R^2^_corr_ = 0.304; *p* < 0.001

In the nHS group (Table 4) the explained variance was smaller as compared to the HS group. The variables included in the model explained 22.6% of the total CPI variance (*R*^*2*^ = 0.275, *R*^*2*^_*corr*_ = 0.226, *F* (3,44) = 5.56, *p* = 0.003). In this group, the level of cognitive complaints was significantly increased if depressive mood was enhanced. Neither age nor the recall performance served as a significant predictor.

**Table 4:** Regression analysis for level of SCI in nHS. *GDS* Geriatric Depression Scale, *FNAT recall* NOS face-name association test recall; significance level: *p < 0.05, **p < 0.01, ***p < 0.001

	B	SE of B	β	p
Constant	2.507	0.335		< 0.001***
Age	− 0.004	0.004	− 0.123	0.376
GDS	0.081	0.022	0.480	0.001**
FNAT recall	− 0.030	0.020	− 0.197	0.150

R^2^ = 0.275, R^2^_corr_ = 0.226; *p* = 0.003

**Figure 3:**
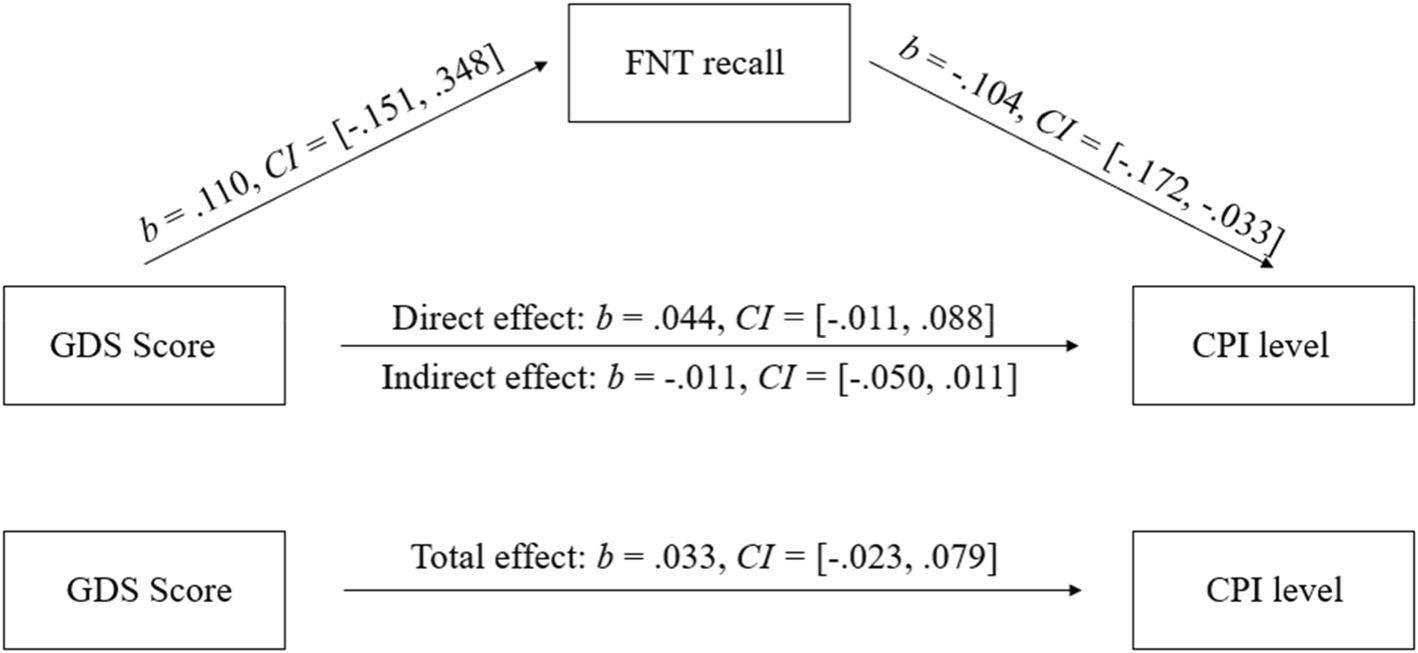
Path of total, direct and indirect effects of the GDS score on the CPI level with FNAT recall (face–name association test recall) included as mediator. *GDS* Geriatric Depression Scale, *CPI* Complainer Profile Identification, *FNAT* face–name association test; Only a significant direct effect of FNAT recall on CPI level was observed. There were no significant total, direct or indirect effects of GDS score on CPI level.

Since a previous study suggested that the predictive value of the performance score reflects a mediator effect^15^, an additional mediation analysis was computed. In the model, the relation between the depressive mood (GDS score) and the level of complaints (CPI level) was tested for a mediation by the recall performance (FNAT recall). The analysis revealed no significant direct effect of GDS on CPI level (*b* = 0.044, *CI* = [− 0.011, 0.088]). Entering the recall performance as a mediator did not lead to a significant indirect effect (*b* = − 0.011, *CI* = [− 0.050, 0.011]). The total effect—based on the sum of direct and indirect effect of GDS on CPI level—was also non-significant (*b* = 0.033, *CI* = [− 0.023, 0.079]). The results are visualized in Fig. 3.

To address the question whether the predictive value of the cognitive performance depends on the choice of the psychometric instrument, we considered the performance of a standard verbal learning test available in the HS group. This additional information was available only in a subset of the HS group (n = 35), and the level of performance is presented in Table 5. The z-standardized age-corrected mean values reveal that the level of performance is slightly better in the standard verbal learning test. The predictive value of both tests was then compared running the regression model formerly applied for the HS group.

**Table 5:** Performance of the HS-group in the FNAT and verbal learning tests. Age-normed z-scores were calculated for each participant in this group. The table shows the *mean* and *SD* of those normed scores. *FNAT* faces-names association test.

	FNAT		Verbal learning test	
	Mean	SD	Mean	SD
Learning rate	− 0.51	1.10	− 0.03	1.14
Recall	− 0.52	1.02	− 0.07	1.04
Recognition	− 0.43	1.30	0.40	0.74

As stated before, performance in the standard verbal learning test and the FNAT recall are correlated (r = 0.42, controlled for age). The results of this regression analysis are depicted in Table 6. The predictors together predicted 44.7% of variance (*R*^*2*^ = 0.512, *R*^*2*^_*corr*_ = 0.447, *F* (4, 30) = 7.87, *p* < 0.001). SCI level was found to be mostly affected by the recall performance in the FNAT score—but not by the recall performance in the verbal ‘gold standard’ tests.

**Table 6:** Regression analysis for level of SCI in a subset of HS: The predictive effects from the scoring in recall performance based in the FNAT and “gold-standard” tests (verbal learning recall) are differently expressed. *GDS* Geriatric Depression Scale, *FNAT* NOS face-name association test recall; significance level: *p < 0.05, **p < 0.01, ***p < 0.001

	B	SE of B	β	p
Constant	4.594	0.579		< 0.001***
Age	− 0.017	0.007	− 0.374	0.022*
GDS	0.049	0.021	0.315	0.027*
FNAT recall	− 0.125	0.035	− 0.580	0.001**
Verbal learning recall	− 0.083	0.085	− 0.138	0.339

R^2^ = 0.512, R^2^_corr_ = 0.447; *p* < 0.001

An additional analysis of this subset (see supplementary data, p.2) included the factor ‘Age’, ‘GDS’, and the ‘Verbal Learning Recall’—but not the FNAT recall: This model only explained only 23.7% of the variance (*R*^*2*^_*corr*_ = 0.237). More importantly, the predictive value of verbal learning (β = − 0.360, p = 0.023) was inferior to that of the psycho-affective state (GDS: β = 0.415, p = 0.012).

## Discussion

In this study, we identified predictors of subjective cognitive impairments (SCI) in two samples of community-dwelling individuals: In help-seekers (HS), recall performance in a face–name association test (FNAT) is closely related to the SCI level. In this group, depressive mood does not serve as a potent and reliable predictor. Moreover, it does not mediate the effect of the mnestic factor. Although the overall-level of depressive mood is less expressed in the group of non-Help-Seekers (nHS), the affective state is a significant predictor for the level of SCI for individuals in this group. Moreover, the results signal that the choice of the psychometric instrument is important: The predictive power of the recall performance observed in HS individuals holds for the FNAT which outperforms the recall score as estimated on the basis of the commonly used verbal learning-and-memory tests. The implication of these results will be discussed in the following.

First, we will focus on the different predictors in the two groups of individuals. Depressive mood was identified as a significant predictor for the level of subjective complaints in previous studies^33,34^. This study confirms that the predictive value of depressive mood is primarily expressed in the group of nHS^15^. Despite a rather low level of depressive mood—also observed in the previous study—the GDS score was closely related to the level of SCI. We like to suggest that the depressive mood in the group of nHS individuals reflects itself in an increased level of general health concerns^35^—which also spreads to the subjective cognitive performance. As highlighted in recent studies^36–38^, this relation might critically depend on personality factors, such as neuroticism which reflects the general tendency to experience negative emotions. A corresponding mediation will have to be explored in future studies.

In an independent sample of nHS individuals (n = 79), data from a verbal learning test (CERAD^32^) were available in addition to the NOS. The corresponding regression analysis revealed that only depressive mood serves as a significant predictor (β = 0.415, p < 0.001), but neither the recall performance in the face–name nor the verbal learning test. This result confirms the regression model for the nHS group (see Table 4), and emphasizes the predictive role of recall performance in the FNAT in the HS group (see Table 6). Please note that this sample has not been matched to the group of HS individuals. The characteristics of the group and the full regression model can be found in the supplement.

Also, in line with previous results^15^, the predictive value of depressive mood is less expressed in the group of help-seeking participants (HS): Here, the impact of the GDS score was reduced (HS: β = 0.188 vs nHS: β = 0.480), and the importance of depressive mood as a predictor of the SCI level can therefore be questioned^19^. Moreover, we can rule out that predictive value of cognitive performance on the level of SCI is due to a mediation effect involving the level of depressive mood. Such a mediation was previously described for attentional tasks^15,22^: In HS individuals, a higher level of depression leads to a reduced processing speed which—in turn—increases the SCI level. The actual study focused on the performance in mnestic tasks, and identified the recall performance as a significant predictor. Our analysis showed that the predictive power of recall performance cannot be explained by a mediation. In other words, cognitive complaints in HS individuals signal mnestic problems—independently of the expression of depressive symptoms. A mechanism—as suggested for nHS—can therefore be ruled out in this group.

However, we have to consider that help-seeking behavior is not only characterized by a problem-focused, planned behavior involving interpersonal interaction^23^—but involves serious health concerns which are frequently related to a higher level of depressive mood. Evidence is provided in our sample, which involved 20 individuals of the HS groups surpassing the GDS cut-off point. Based on previous findings^39^, we propose that the higher level of depressive mood contributes to a more realistic self-assessment of cognitive abilities and that this process increases the validity of the rating of cognitive problems in daily living provided in the questionnaire.

The higher level of depressive mood in HS individuals might not only affect the evaluation of the own cognitive abilities, but also the level of cognitive performance. Previous studies already indicated that problems in episodic memory can be frequently encountered in depressive illness^40^. We like to propose that these problems are more validly registered in a questionnaire related to problems in daily living, and that they can be uncovered in the face–name association test.

In sum, we assume a higher sensitivity for cognitive problems in the group of help-seekers which is due to the elevation in health concerns and depressive mood. This predisposition might contribute to problems in episodic memory—but does not determine the level of SCI. The latter can be primarily related to the performance in the psychometric tests.

Second, our data indicate that the choice of the neuropsychological test procedure is important. The high predictive value of the recall performance on the general level of SCI has not been congruently observed in earlier studies: Mnestic performance was either embedded in a compound score^41^, or other cognitive domains served as superior predictors^15,18^. The predictive value of the recall performance identified in this study depends critically on the material used in the psychometric test: The recall of word lists—commonly used in verbal learning-and-memory tests—depends on the application of cognitive strategies in the acquisition phase, and therefore on the cognitive reserve^42^. Despite of limitations in episodic memory, HS individuals can frequently rely on their cognitive reserve, and the associated compensatory activation has already been reported^43^. Accordingly, our data show that recall performance as predicted by means of verbal learning tests does not provide a primary predictor for the SCI level, and its predictive value is outperformed by the psycho-affective state.

In contrast, the FNAT represents a cognitively more-demanding task^28^: Faces are associated with less context cues as compared to words and the assignment to the personal name is more or less random^44^. Moreover, personal names cannot be categorized and integrated in existing semantic knowledge as easily as words^45^. Consequently, the learning of face–name associations primarily relies on massed repetition^46^. This results in a test score which is less affected by compensatory strategies and contributes to a high discriminant validity of the recall performance as measured in the FNAT and the standard tests (CERAD, VMT). Most importantly, the more-restrictive FNAT score uncovers problems in episodic memory which are also remarked by the help-seeking individuals.

The relation between FNAT recall score and the SCI is also enhanced by the relevance of retrieving face–name associations in daily living: Forgetting names commonly leads to high embarrassment^47^ and corresponding episodes can be assumed to remain active in autobiographical memory. We tentatively suggest that the level of subjective complaints in HS individuals is significantly affected by the frequency of these events.

Our conclusions are limited concerning the generalizability by the following factors: (1) We have to consider the skewness in the distribution of age in our sample: Following the matching process between the groups, only 5–6 participants in each group were older than 70 years. This effect might contribute to the—unexpected—direction of the predictor ‘age’: In contrast to earlier studies^48^, the SCI level was more enhanced in younger participants. One might assume that older individuals tend to attribute their cognitive problems to a normal aging process^49^. This complex interaction remains to be explored. (2) The registration of the mnestic performance did not involve a valid estimation of working memory performance. Although previous clinical studies indicate a high sensitivity of the visual binding test^50^, the majority of the individuals in our sample performed at ceiling. Using the binding performance as a predictor will therefore require an increase in task difficulty, e.g. by enhancing the number of items to be remembered. (3) The standard neuropsychological measurement (including the verbal learning-and-memory test) was only applied in the HS group. Within our matched sample of nHS, we cannot rule out that the performance in a verbal learning test provides a predictor for the level of SCI. However, the data presented for the non-matched nHS group (n = 79) provide strong evidence that a relation between verbal recall (words and face–names) and subjective memory complaints is unlikely. (4) Although the association of a face and a personal name is random, and learning is supposed to rely on massed repetition^46^, we cannot rule out that individuals used metacognitive strategies. Forthcoming studies using the FNAT, will therefore also consider a retrospective report of the individuals on the strategies used. (5) Finally, the size and composition of our sample must be considered: Irrespective of the power analysis, the sample size (n = 96) appears to be rather small. However, the trustworthiness of the results is increased by the fact that the crucial interaction in the regression analysis (see Table 2) is a replication of previous findings in an independent sample^15^. The generalizability of the findings is rather reduced by fact that most participants can be related to a western, well-educated, industrialized, rich, and democratic (WEIRD) social background^51^. In this sample, help-seeking behavior might be differently expressed as compared to other groups of different socio-economic background.

In conclusion, our results show that self-reported cognitive impairment in help-seekers can be related to mnestic problems. Although depressive symptoms are more frequent in this sample, the relation between subjective complaints and objective performance does not depend on the psycho-affective state of the help-seeker. Most importantly, the relation can be uncovered if the test instrument reduces the effect of compensatory strategies.

The clinical use of the online screening procedure presented will have to be examined in further studies. Neuroimaging techniques might answer the question whether the recall problems in help-seekers can be related to neuropathological conditions recently reported in SCI patients^52^. In addition to a follow-up approach, the online tool might contribute to an early identification of individuals with a higher risk for developing a neurodegenerative disease^3,53^.

## Methods

### Participants

This study was conducted in accordance with relevant guidelines and regulations and the study protocol was approved by the ethics committee of FU Berlin (No. 011/2022). All participants signed informed consent. An a priori power analysis (G*Power^54^) on the base of a linear multiple regression (f = 0.15, alpha = 0.05, power = 0.80, predictors = 5) revealed that at least 92 participants are needed to be included. 277 participants completed the NOS. Of those 166 were healthy controls, while 61 participants actively sought help for SCI in the neuropsychological consultation hour of FU Berlin. This latter group is described as help seekers (HS), while the healthy controls compose the non-help-seeker group (nHS). Non-help-seekers (nHS) were recruited via public information events at the FU Berlin. Matching partners for HS individuals were identified out of this sample. In a sub-sample of healthy controls (n = 79), psychometric data from a verbal learning test (CERAD^32^) were collected. We matched one nHS to each HS based on gender, age and education. Education was measured as three categories according to the German school diplomas: *Hauptschule/POS* 8th/9th grade (10 years or less of basic education), *Realschule/POS* 10th grade (10 years of schooling), and *Hochschulreife* (12 years or more of schooling). Matching was done through propensity scores with the nearest neighbor method without replacement and a caliper of 0.25 SD of the logit of the propensity score. After matching, the sample was composed of 96 participants with 48 subjects in both the HS- and the nHS-group. The matched sample showed a good balance for the standardized mean differences and the variance ratios. The mean age of the sample is 51.70 years (*SD* = 14.70 years) with an age range between 18 and 87 years old. 50 participants were female, 46 male. To approach the socioeconomic status, we considered education and employment status. Education already served as a matching criterion. With respect to employment status (unemployed vs employed vs retired vs student status), no differences in frequencies were obtained between the groups (see Table 7).

**Table 7:** Descriptive and inference statistics for employment status per group. *nHS* non-help-seekers, *HS* help-seekers; significance level: *p < 0.05, **p < 0.01, ***p < 0.001.

Employment status	Unemployed	Employed	Retired	Student
nHS	1	36	10	1
HS	5	29	11	3

*χ*^*2*^ (3*, N* = 96) = 4.468, *p* = 0.215

### Stimuli and procedure

Participants in both groups were sent a link to the online platform Pavlovia (https://run.pavlovia.org/nelep92/kos) and completed the NOS (CPI, GDS, FNAT, and visual short-term memory binding task) at home. HS individuals had an appointment in the neuropsychological consultation hour at the FU Berlin, and underwent an additional individual in-person examination. The ‘Complainer Profile Identification’ (CPI) was completed before all objective measures to ensure that rating of SCI was not confounded with perceived performance in those tests.

The single components of the NOS will be described in detail in the following.

*CPI:* The ‘Complainer Profile Identification’ questionnaire^13^ was used to measure SCI. The questionnaire is composed of 17 items regarding subjective impairment in three cognitive domains: attention, memory and executive functioning. The items are related to problems in daily function to increase the ecological validity. An example item for the memory domain is: “I recently have to look up phone numbers, which I used to know by heart.” All items are to be rated on a 5-point rating scale with respect to the frequency of the problems in daily life (from 1 = never to 5 = very often). For the statistical analysis, a mean was extracted for each participant. The complete questionnaire can be found in the supplementary material page 7.

*GDS* (Geriatric Depression Scale): Depressive mood was measured using a short version of the GDS^29^. Importantly, this study used the GDS as a measure for depressive mood only, not for a clinical depression. The short version of GDS consists of 15 items that can be answered with yes or no (e.g. “Do you feel that your life is empty.”). The final individual score reflects the number of answers given that indicate depressive mood. The GDS was chosen specifically for its use in the elderly because we expected older people to be the main target group for the neuropsychological consultation hour and therefore composing a large part of the HS-group.

*FNAT* (Face-name association test): In this test, participants are required to learn and retrieve face–name associations. For this, two sets of 12 photorealistic faces were selected from a collection of computer-generated faces^55^. The selected faces were rated by four independent raters as being realistic and covered an age range from approximately 18 to 75 years. Half of each set consists of male and the other half of female faces. Both sets were matched with respect to the similarity of each pair of faces (sex, age, global characteristics). To increase familiarity, faces were matched to social environment of most help seekers of the neuropsychological consultation hour (Caucasian, no tattoos or piercing). To each face a first name of high frequency was assigned.

The procedure for the FNAT can be seen in Fig. 1. In the initial study phase (see Fig. 1: T1), participants were shown the 12 faces and their assigned names from the first set one after another. Each face–name association was presented in randomized order for three seconds. After each study phase, the faces were presented again and the participant was asked to enter the first letter of the corresponding name or an X if they do not remember the name. The study and test phase were repeated three times (learning phase). Next, an interference list with the 12 faces from the second—matched—set was studied and tested in the same way the first set was (see Fig. 1: I). After the interference test, another free recall test phase of the first set of face–name associations is commenced (see Fig. 1: T4). To introduce a temporal delay of approximately 15–20 min, participants complete a short-term memory binding test (see below). Then, the faces of the first list were presented again, and the first letter of the corresponding name was to be entered. The delayed recall test (see Fig. 1: T5) serves as a sensitive predictor of age-related memory problems, but has also been related to neurodegenerative disease^56^. Lastly, the participants completed a recognition task (see Fig. 1: R). For the recognition task each face of the first set was presented with four age-appropriate first names, of which one is the correct corresponding first name. Of these four names two names each start with the same letter. This way, participants have to remember the full name correctly to finish this test and not only the first letter.

For each participant, scores for three consecutive learning trials (trial 1, trial 2, trial 3), an interference list (interference), two recall episodes (recall, delayed recall), and the final recognition procedure (recognition) were extracted.

*Short term memory binding task*: The NOS furthermore includes a visual short term-memory binding task which has been established in the neuropsychological diagnostics of neurodegenerative processes^57^. Following the setup developed by Parra and colleagues^58^, our task involves three blocks each comprising 64 trials. In each trial, two successive images each containing two items are presented. In the first block, participants have to decide on the match of the color of the items in a two-alternative-forced-choice (2-AFC) procedure. In the second block, the form of the items has to be matched. In the third block, participants have to decide whether the assignment of form and color of the items has been swapped in the two successive images (binding condition). The setup is described in more detail in the supplementary material (page 8). For the Binding test, the discrimination index *A*′ was calculated according to^59^. Since the participants performed at ceiling in this task, the data were not considered in the following analysis.

Participants in the HS group sought help in the neuropsychological consultation hour of FU Berlin, and were additionally examined with standardized neuropsychological tests. In 35 (out of 47) participants, this examination also included a verbal memory test. Depending on the age of the participant, either the VLMT (Verbaler Lern- und Merktest^31^) or the CERAD (Consortium to Establish a Registry for Alzheimer’s Disease^32^) was applied. Comparable to the FNAT, the tests provide separated test values for the learning phase, the recall and recognition.

### Statistical analysis

For each participant, GDS score, overall CPI level and performance scores were calculated. Group differences regarding gender, education level, age, CPI level, GDS score and the cognitive performance scores were tested for by running Chi-Squared-tests and ANOVAs, respectively.

The analysis related to the first research question requires a linear multiple regression analysis for the dependent variable CPI level. General assumptions were met. In a first step, a stepwise regression was applied to select relevant predictors.

The following predictors were considered: age, GDS score, FNAT trial 1, FNAT proactive interference, FNAT recall, as well as group (HS vs. nHS) as a moderator. The following scores were not considered to reduce the number of predictors in the regression model: The FNAT trial 2 and 3 were highly corelated with the FNAT trial 1 score. The same accounts for the FNAT delayed recall score which was highly correlated with FNAT recall. For the FNAT recognition the majority of individuals performed at ceiling (77%). This also accounts for the binding performance (discrimination index A′ = 72%). The stepwise regression returned group, age, GDS score and FNAT recall as useful predictors. The results of the initial regression analysis are provided in the supplementary data, page 9.

To test for the first hypothesis, the predictors previously identified were entered. The model also included the relevant interaction of group assignment, GDS score, and FNAT recall performance. The variables were entered in a linear multiple regression analysis. As the interactions turned out to be significant, separate regression analysis for the two groups were computed. In each regression analysis (SCI level in HS, SCI level in nHS), the variables “age”, “GDS score”, and “FNAT recall” were entered. Finally, we calculated a mediation model in the HS-group to test whether the effect of depressive symptoms on CPI level is mediated by recall performance. Running the R module lavaan, indirect effects were tested for by using estimate bias-corrected bootstrap mediation analysis^60^ confidence intervals and 1000 bootstrapping samples.

To compare the predictive value of recall performance as estimated by different psychometric tests, the “FNAT recall” score and the “standard-verbal-learning-test” score were entered in a linear multiple regression analysis for CPI level. Since the FNAT recall scores were not age corrected but the verbal learning test scores were, age was also considered in the regression model. Finally, the GDS score was considered to allow comparison with the previous models.

The analysis were carried out with IBM SPSS Statistics 27^61^ and R 4.1.2. using RStudio^62^.

### Supplementary Information

Supplementary Information.

## Data Availability

The data that support the findings of this study are openly available in OSF repository at https://osf.io/k6wv9/?view_only=c12ca946cebc4411a605621f7a549a26.

## References

[1] Jessen F (2014). A conceptual framework for research on subjective cognitive decline in preclinical Alzheimer's disease. Alzheimers Dement..

[2] Röhr S (2020). Estimating prevalence of subjective cognitive decline in and across international cohort studies of aging: A COSMIC study. Alzheimer's Res. Ther..

[3] Wang XT (2021). Association of subjective cognitive decline with risk of cognitive impairment and dementia: A systematic review and meta-analysis of prospective longitudinal studies. J. Prev. Alzheimer's Dis..

[4] Frankenmolen NL (2017). Memory strategy use in older adults with subjective memory complaints. Aging Clin. Exp. Res..

[5] Ibnidris A (2022). Evaluating measurement properties of subjective cognitive decline self-reported outcome measures: A systematic review. Syst. Rev..

[6] Müller-Gerards D (2019). Subjective cognitive decline, APOE ε4, and incident mild cognitive impairment in men and women. Alzheimer's Dement..

[7] van Wanrooij LL, Richard E, Jongstra S, Moll van Charante EP, van Gool WA (2019). Associations of subjective memory complaints and simple memory task scores with future dementia in the primary care setting. Ann. Fam. Med..

[8] Abdulrab K, Heun R (2008). Subjective Memory Impairment A review of its definitions indicates the need for a comprehensive set of standardised and validated criteria. Eur. Psychiatry.

[9] Graham NL, Emery T, Hodges JR (2004). Distinctive cognitive profiles in Alzheimer’s disease and subcortical vascular dementia. J. Neurol. Neurosurg. Psychiatry.

[10] Chipi E (2018). The Italian version of cognitive function instrument (CFI): Reliability and validity in a cohort of healthy elderly. Neurol. Sci..

[11] La Joie R (2016). Qualitative and quantitative assessment of self-reported cognitive difficulties in nondemented elders: Association with medical help seeking, cognitive deficits, and β-amyloid imaging. Alzheimer's Dement..

[12] Beblo T, Kunz M, Brokate B, Scheurich A, Weber B, Albert A, Richter P, Lautenbacher S (2010). Construction of a questionnaire for complaints of cognitive disturbances in patients with mental disorders. Z. Neuropsychol..

[13] Lubitz AF, Eid M, Niedeggen M (2018). Complainer Profile Identification (CPI): Properties of a new questionnaire on subjective cognitive complaints. Aging Neuropsychol. Cogn..

[14] Youn JC, Kim KW, Lee DY, Jhoo JH, Lee SB, Park JH, Choi EA, Choe JY, Jeong JW, Choo IH, Woo JI (2009). Development of the subjective memory complaints questionnaire. Dement. Geriatr. Cogn. Disord..

[15] Lubitz AF, Eid M, Niedeggen M (2020). Psychosocial and cognitive performance correlates of subjective cognitive complaints in help-seeking versus non-help-seeking community-dwelling adults. J. Geriatr. Psychiatry Neurol..

[16] Stogmann E (2015). Activities of daily living and depressive symptoms in patients with subjective cognitive decline, mild cognitive impairment, and Alzheimer’s disease. J. Alzheimers Dis..

[17] Lucas HD (2016). Relational memory and self-efficacy measures reveal distinct profiles of subjective memory concerns in older adults. Neuropsychology.

[18] Rouch I (2008). Cognitive complaints, neuropsychological performance and affective disorders in elderly community residents. Disabil. Rehabil..

[19] Burmester B, Leathem J, Merrick P (2016). Subjective cognitive complaints and objective cognitive function in aging: A systematic review and meta-analysis of recent cross-sectional findings. Neuropsychol. Rev..

[20] Zlatar ZZ, Muniz M, Galasko D, Salmon DP (2018). Subjective cognitive decline correlates with depression symptoms and not with concurrent objective cognition in a clinic-based sample of older adults. J. Gerontol. B-Psychol..

[21] Chin J, Oh KJ, Seo SW, Na DL (2014). Are depressive symptomatology and self-focused attention associated with subjective memory impairment in older adults?. Int. Psychogeriatr..

[22] Soellner A, Lubitz AF, Lueschow A, Niedeggen M (2021). Predictors of subjective cognitive impairment in help-seekers with and without symptoms of a major depression. GeroPsych.

[23] Cornally N, McCarthy G (2011). Help-seeking behaviour: A concept analysis: Help-seeking behaviour: A concept analysis. Int. J. Nurs. Pract..

[24] Jessen F (2020). The characterisation of subjective cognitive decline. Lancet Neurol..

[25] Contemori G, Saccani MS, Bonato M (2022). Multitasking effects on perception and memory in older adults. Vision (Basel).

[26] Weissberger GH (2017). Diagnostic accuracy of memory measures in Alzheimer’s dementia and mild cognitive impairment: A systematic review and meta-analysis. Neuropsychol. Rev..

[27] Spooner D, Pachana N (2006). Ecological validity in neuropsychological assessment: A case for greater consideration in research with neurologically intact populations. Arch. Clin. Neuropsychol..

[28] Rentz DM (2011). Face-name associative memory performance is related to amyloid burden in normal elderly. Neuropsychologia.

[29] Yesavage JA, Sheikh JI (1986). 9/Geriatric Depression Scale (GDS): Recent evidence and development of a shorter version. Clin. Gerontol..

[30] Kurlowicz L, Greenberg SA (2007). The Geriatric Depression Scale (GDS). Am. J. Nurs..

[31] Helmstaedter, C., Lendt, M. & Lux, S. VLMT—Verbaler Lern- und Merkfähigkeitstest. *Belitz Test GMBH* (2001).

[32] Moms JC, Heyman A, Mohs RC, Hughes JP, van Belle G, Fillenbaum G, Mellits ED, Clark C (1989). The Consortium to Establish a Registry for Alzheimer’s Disease (CERAD). Part I. Clinical and neuropsychological assessment of Alzheimer’s disease. Neurology.

[33] Reid LM, MacLullich AMJ (2006). Subjective memory complaints and cognitive impairment in older people. Dement. Geriatr. Cogn. Disord..

[34] Zlatar ZZ, Moore RC, Palmer BW, Thompson WK, Jeste DV (2014). Cognitive complaints correlate with depression rather than concurrent objective cognitive impairment in the successful aging evaluation baseline sample. J. Geriatr. Psychiatry Neurol..

[35] Redelmeier DA, Najeeb U, Etchells EE (2021). Understanding patient personality in medical care: Five-factor model. J. Gen. Intern. Med..

[36] Bell T, Hill N, Stavrinos D (2020). Personality determinants of subjective executive function in older adults. Aging Ment. Health.

[37] Pearman A, Hertzog C, Gerstorf D (2014). Little evidence for links between memory complaints and memory performance in very old age: Longitudinal analyses from the Berlin Aging Study. Psychol. Aging.

[38] Smit D, Koerts J, Bangma DF, Fuermaier ABM, Tucha L, Tucha O (2021). Look who is complaining: Psychological factors predicting subjective cognitive complaints in a large community sample of older adults. Appl. Neuropsychol. Adult.

[39] Moore MT, Fresco DM (2012). Depressive realism: A meta-analytic review. Clin. Psychol. Rev..

[40] Airaksinen E, Larsson M, Lundberg I, Forsell Y (2004). Cognitive functions in depressive disorders: Evidence from a population-based study. Psychol. Med..

[41] Amariglio RE, Townsend MK, Grodstein F, Sperling RA, Rentz DM (2011). Specific subjective memory complaints in older persons may indicate poor cognitive function: Memory complaints and objective testing. J. Am. Geriatr. Soc..

[42] Darby RR, Brickhouse M, Wolk DA, Dickerson BC (2017). Effects of cognitive reserve depend on executive and semantic demands of the task. J. Neurol. Neurosurg. Psychiatry.

[43] Zhang Z (2021). Changes of regional neural activity homogeneity in preclinical Alzheimer’s disease: Compensation and dysfunction. Front. Neurosci..

[44] Werheid K, Clare L (2007). Are faces special in Alzheimer's disease? Cognitive conceptualisation, neural correlates, and diagnostic relevance of impaired memory for faces and names. Cortex.

[45] Hirst W, Volpe BT (1988). Memory strategies with brain damage. Brain Cogn..

[46] Mangels JA, Manzi A, Summerfield C (2010). The first does the work, but the third time's the charm: The effects of massed repetition on episodic encoding of multimodal face–name associations. J. Cogn. Neurosci..

[47] Hargis MB, Whatley MC, Castel AD (2020). Remembering proper names as a potential exception to the better-than-average effect in younger and older adults. Psychol. Aging.

[48] Markova H (2017). Subjective cognitive complaints in cognitively healthy older adults and their relationship to cognitive performance and depressive symptoms. J. Alzheimers Dis..

[49] Ponds WHM, van Boxtel MPJ, Jolles J (2000). Age-related changes in subjective cognitive functioning. Educ. Gerontol..

[50] Pietto M (2016). Behavioral and electrophysiological correlates of memory binding deficits in patients at different risk levels for Alzheimer’s disease. J. Alzheimers Dis..

[51] Henrich J, Heine SJ, Norenzayan A (2010). The weirdest people in the world?. Behav. Brain Sci..

[52] Ortega G (2021). Combination of white matter hyperintensities and Aβ burden is related to cognitive composites domain scores in subjective cognitive decline: The FACEHBI cohort. Alzheimer's Res. Ther..

[53] Pike KE, Cavuoto MG, Li L, Wright BJ, Kinsella GJ (2021). Subjective cognitive decline: Level of risk for future dementia and mild cognitive impairment, a meta-analysis of longitudinal studies. Neuropsychol. Rev..

[54] Erdfelder, E. *et al.* GPOWER: A general power analysis program. *Behav. res. meth. instrum. comput.* **28**, 1–11 (1996).

[55] Karras, T., Laine, S., Aittala, M., Hellsten, J., Lehtinen, J. & Aila, T. *This Person does not exist.* https://thispersondoesnotexist.com/ (2019).

[56] Gainotti G, Quaranta D, Vita MG, Marra C (2013). Neuropsychological predictors of conversion from mild cognitive impairment to Alzheimer's disease. J. Alzheimers Dis..

[57] Martínez JF, Trujillo C, Arévalo A, Ibáñez A, Cardona JF (2019). Assessment of conjunctive binding in aging: A promising approach for Alzheimer’s disease detection. J. Alzheimers Dis..

[58] Parra MA, Abrahams S, Logie RH, Sala SD (2009). Age and binding within-dimension features in visual short-term memory. Neurosci. Lett..

[59] Isella V, Molteni F, Mapelli C, Ferrarese C (2015). Short term memory for single surface features and bindings in ageing: A replication study. Brain Cogn..

[60] MacKinnon DP (2008). Introduction to Statistical Mediation Analysis.

[61] IBM Corp. IBM SPSS Statistics for Windows, Version 27.0 (2020).

[62] RStudio: Integrated Development for R. RStudio, Inc (2019).

