# Supplementary material for: Uncovering mnestic problems in help-seeking individuals reporting subjective cognitive complaints
Source: Sci Rep. 2023 Sep 14;13:15266. doi: 10.1038/s41598-023-42527-x (PMC10502030; doi:10.1038/s41598-023-42527-x)
Supplement: Supplementary file 1 — Supplementary Information. [file 41598_2023_42527_MOESM1_ESM.pdf]

Supplementary Material

Contents

Regression analysis for level of SCI based on age, depressiveness and verbal learning recall . 2

Regression analysis for level of SCI based on age, depressiveness and FNAT recall and verbal learning recall 3

Histograms for GDS 5

CPI: Complainer Profile Identification 7

NOS: Short-term memory binding test (STMB) 8

Results of the stepwise-regression to determine appropriate predictors 9

Regression analysis for level of SCI based on age, depressiveness and verbal learning recall

In an analysis of the subset of help-seekers with available performance data for verbal learning recall, we included the factor ‘Age’, ‘GDS’, and the ‘Verbal Learning Recall’ – but not the FNAT recall: Here, the verbal learning recall served as a predictor (ß=- .360, p=.023) - but it was inferior to the psycho-affective state (GDS: ß=.415, p=.012). For the full analysis please refer to supplementary table 1.

Supplementary Table 1

	B	SE of B	β	p
Constant	3.014	.440		<.001***
Age	-.004	.007	-.088	.574
GDS	.064	.024	.415	.012*
Verbal learning recall	-.215	.090	-.360	.023*
R^2 = .304, R^2_{corr} = .237; *p* = .010				

Supplementary Table 1. Regression analysis for SCI based on age, depressiveness and verbal learning recall in a subset of HS. GDS: Geriatric Depression Scale; significance level: p<.05*, p<.01**, p<.001***

Regression analysis for level of SCI based on age, depressiveness and FNAT recall and verbal learning recall

In an unmatched sample of nHS (n=79) results for verbal learning recall were available. The descriptive data for this sample compared to the HS- and matched nHS-group can be seen in supplementary table 2.

Supplementary Table 2

	HS		nHS matched to HS		nHS unmatched group	
	mean	SD	mean	SD	mean	SD
Gender	26 female, 22 male		24 female, 24 male		42 female, 37 male	
Education	<10 years: 6,<br>10 years: 14,<br>>10 years: 28		<10 years: 8<br>10 years: 16<br>>10 years: 24		<10 years: 6<br>10 years: 21<br>>10 years: 52	
Age (in years)	50.63	14.16	52.77	15.29	59.81	9.05
CPI	3.24	0.73	2.27	0.48	2.10	0.47
GDS	7.85	3.98	2.62	2.86	1.75	2.24
FNAT trial 1-3	19.56	8.01	22.71	7.56	23.16	6.97
FNAT interference	4.00	2.68	5.48	2.73	5.67	2.72
FNAT recall	6.71	3.19	8.17	3.19	8.65	2.79
FNAT delayed recall	7.06	3.35	8.19	3.17	8.56	3.00
FNAT recognition	9.79	2.54	10.48	1.95	11.01	1.33

Supplementary Table 2. Descriptive statistics for the HS, nHS (sample matched to HS-group) and an unmatched nHS group (n = 79). CPI: Complainer Profile Identification; GDS: Geriatric Depression Scale; FNAT: Face-Name-Association Test; Verbal learning test used: CERAD word list.

A linear multiple regression analysis revealed: depressive mood is the only significant predictor in this group. Neither recall score can reach significance as a predictor. For exact results please refer to supplementary table 3.

Supplementary Table 3

	B	SE of B	β	p
Constant	2.627	0.546		<.001***
Age	-0.005	0.006	-.097	.416
GDS	0.087	0.023	.415	<.001***
FNAT recall	-0.017	0.020	-.101	.392
Verbal learning recall	-0.028	0.030	-.109	.350
$R^2 = .236$, $R^2_{corr} = .195$; $p < .001$				

Supplementary Table 3. Regression analysis for level of SCI in an unmatched sample of n=79 nHS: The predictive effects from the scoring in recall performance based in the FNAT and "gold-standard" tests (verbal learning recall from CERAD) are differently expressed. GDS: Geriatric Depression Scale; FNAT: NOS faces-names-test recall; significance level: p<.05*, p<.01**, p<.001***

Histograms for GDS

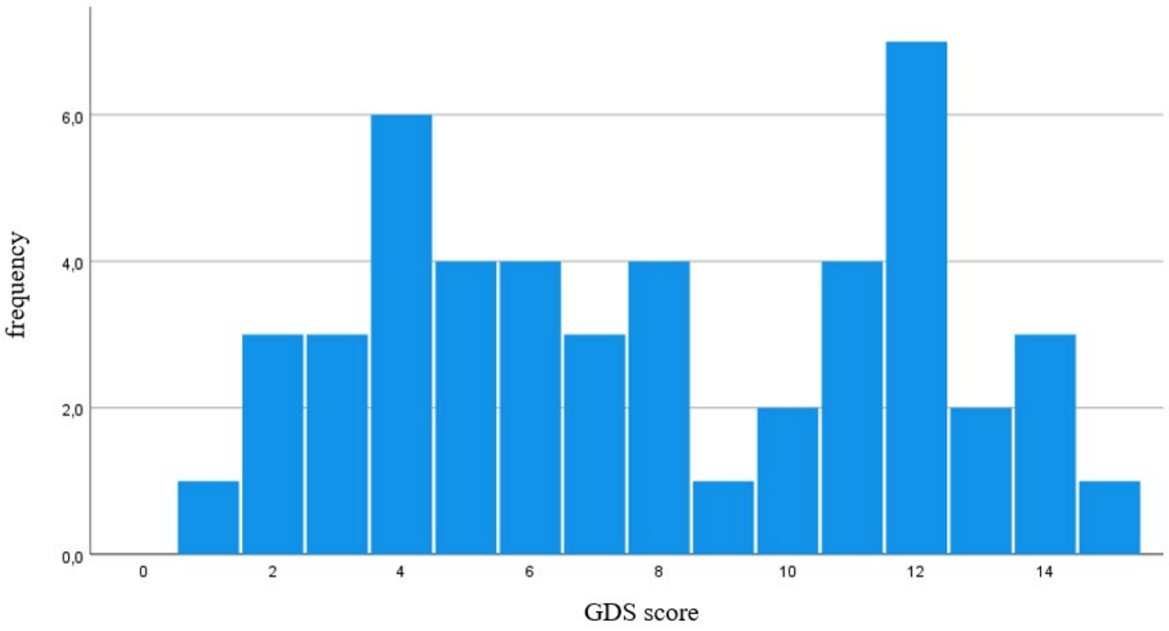

Supplementary Figure 1

Supplementary Figure 1. Distribution of the GDS score in the HS sample. GDS: Geriatric Depression Scale

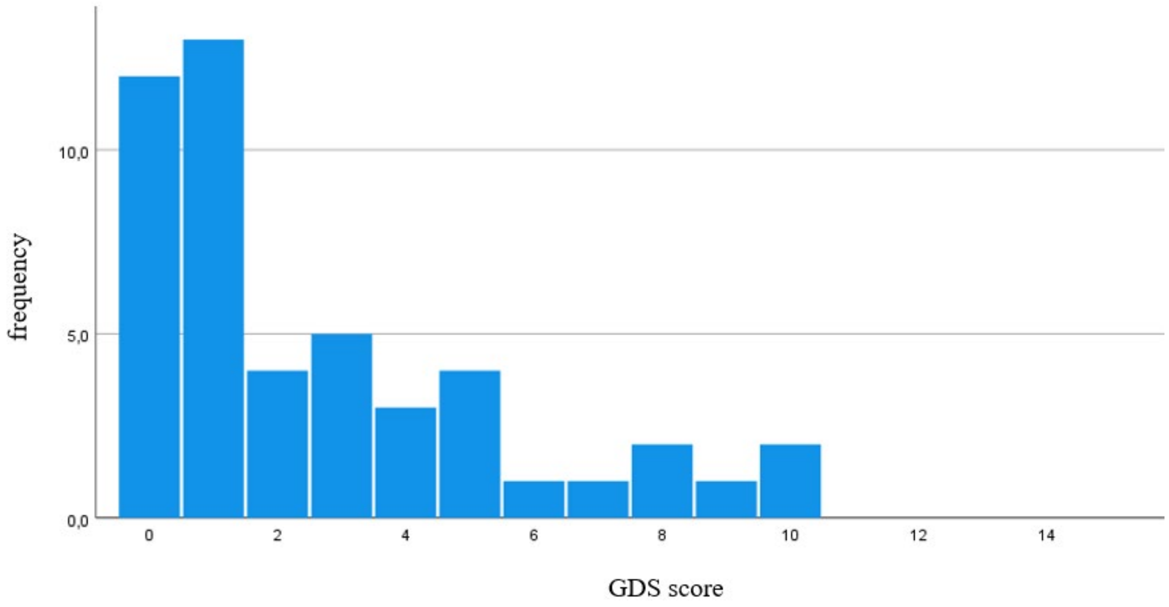

Supplementary Figure 2

Supplementary Figure 2. Distribution of the GDS score in the nHS sample. GDS: Geriatric Depression Scale

Scatter plot for association between age and CPI

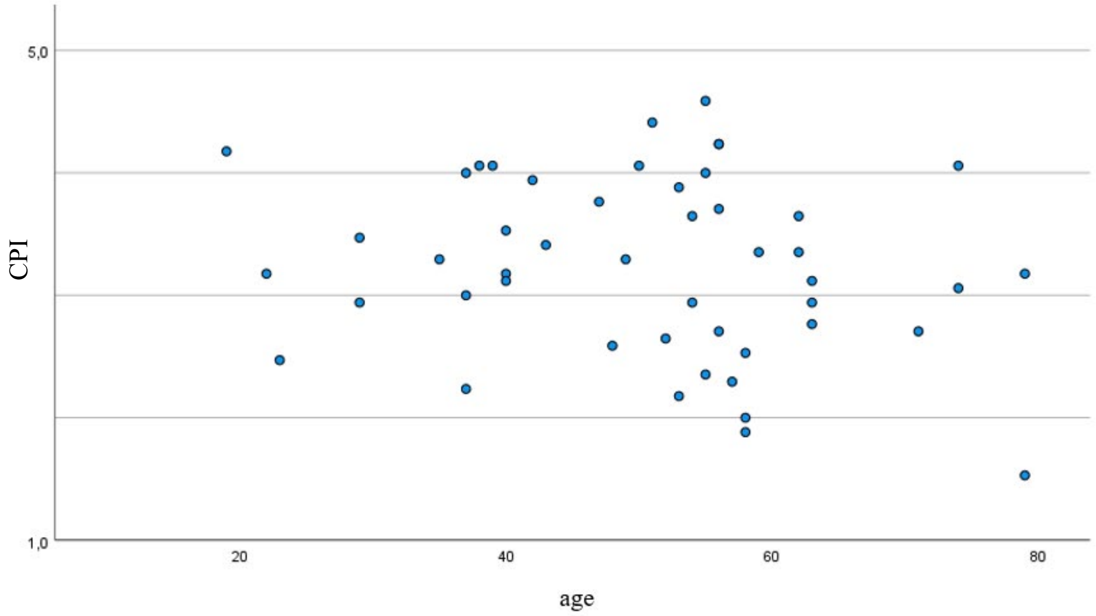

Supplementary Figure 3

Supplementary Figure 3. Scatter plot for the association between age and CPI for the HS sample. CPI: Complainer Profile Identification; age in years

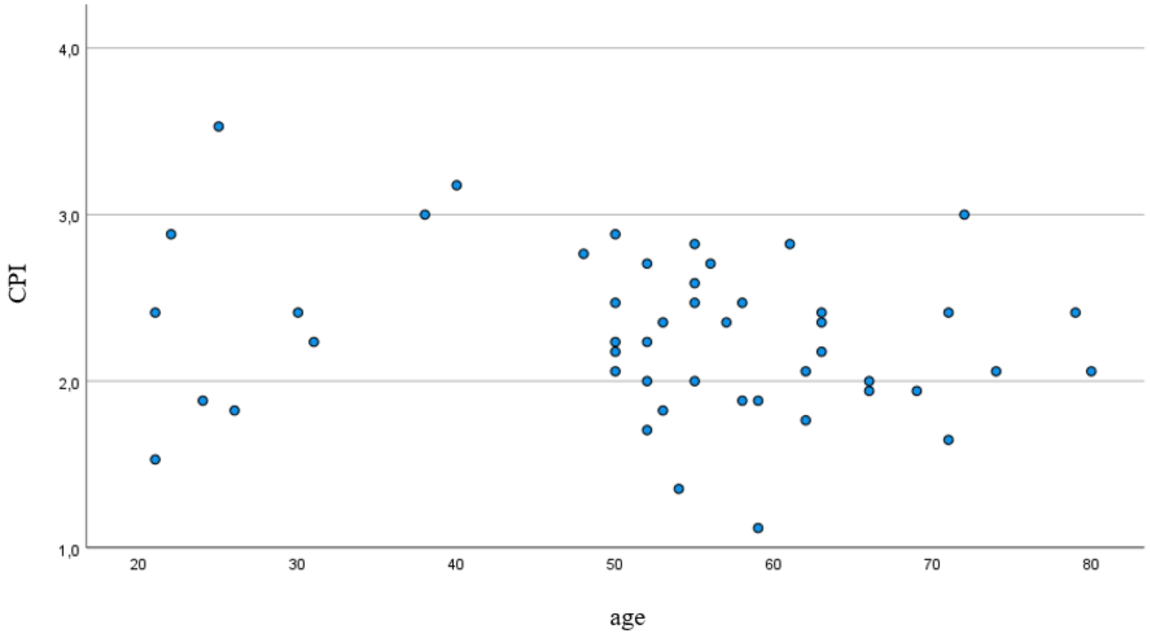

Supplementary Figure 4

Supplementary Figure 4. Scatter plot for the association between age and CPI for the nHS sample. CPI: Complainer Profile Identification; age in years

CPI: Complainer Profile Identification

For the CPI the following situations are rated on a five-point scale on often they occur:

1 = ”never”, 2 = “rarely”, 3 = “sometimes”, 4 = “often”, 5 = “very often”

Supplementary Table 4

German	English
1. Wenn ich eine Tätigkeit ausführe, lasse ich mich schnell von anderen Dingen ablenken.	1. When doing something, I am easily distracted.
2. Es fällt mir schwerer als sonst, zwei Dinge gleichzeitig zu erledigen (z.B. Kochen und Nachrichten hören).	2. It is harder than usual to do two things at once (e.g. cooking and listening to the news).
3. Ich ermüde schneller beim Lesen oder beim Fernsehen.	3. I get tired quicker while reading or watching TV.
4. Ich muss länger nach Gegenständen suchen (z.B. Schlüssel) muss.	4. I have to look for objects (e.g. keys) longer.
5. Ich bemerke, dass ich alltägliche Verrichtungen langsamer ausführe.	5. I notice that I am doing daily activities slower.
6. An frühere Ereignisse (z.B. Urlaube, Geburtstagsfeiern) kann ich mich nur lückenhaft erinnern.	6. I have trouble remembering past events (e.g. vacations, birthday parties).
7. Mir früher bekannte Telefonnummern, muss ich in letzter Zeit nachschauen.	7. Recently, I have to look up telephone numbers that I used to know by heart.
8. Wenn mir jemand etwas sagt, muss ich die Information sofort notieren, damit ich sie nicht vergesse.	8. When someone tells me something, I have to write it down so I don’t forget it.
9. An den Artikel, den ich morgens in der Zeitung gelesen habe, kann ich mich am Nachmittag nicht mehr erinnern.	9. The article that I read in the morning I cannot remember in the afternoon.
10. Den Gesichtern von Personen, die mir vorgestellt worden sind, kann ich später den richtigen Namen nicht zuordnen.	10. When I get to know new people, I cannot match the faces with the correct names later.
11. Ich komme in der zeitlichen Reihenfolge von Ereignissen durcheinander.	11. I confuse the timely order of events.
12. Mir fällt es zunehmend schwerer, komplizierte Sätze zu verstehen.	12. It gets progressively harder to understand complicated sentences.
13. Mir fallen Wörter, die ich sagen will, nicht mehr ein.	13. I don’t remember words that I want to say.
14. Ich muss häufiger überlegen, wie ein Wort geschrieben wird (auch bei einfachen Wörtern).	14. I often have to think about how a word is spelled (even for simple words).
15. Ich habe Probleme, eine Aufgabe zu Ende zu bringen, da ich mehrere Tätigkeiten gleichzeitig beginne.	15. I have trouble finishing a task because I start multiple tasks at once.
16. Ich fühle mich überfordert, wenn etwas nicht routinemäßig verläuft und ich mich auf neue Bedingungen einstellen	16. I often feel overwhelmed when something does not go over routinely or when I have to adapt to new circumstances.
17. Wenn ich sehr viel zu erledigen habe, fällt es mir schwer, dies zu koordinieren	17. I have trouble coordinating tasks when I have a lot on my plate.

Supplementary Table 4. Full Complainer Profile Identification questionnaire in German (original) and English translation.

NOS: Short-term memory binding test (STMB)

In the STMB, an array of two items is shown for 2 seconds in the study phase. The participant is instructed to memorize this stimulus. Then the stimulus disappears and a fixation cross is presented for 1.5 seconds. After that the test array is shown. The test array can consist of the same two items or different items and the participant has to indicate via pressing a key on the keyboard or clicking on a button whether the items are the same or different from the study array. Then the next study array is presented.

The STMB task consists of three conditions: the color-condition, the form-condition and the binding- or color-form-condition. In the color-condition the items are two shapes in two different colors and in the test array the colors might be different. In the form-condition the two items are two different black geometric shapes and in the test array the shapes might be different. In the binding condition the study array consists of two shapes in a color each. In the test array, the shapes might have switched colors and differ in that way from the study array.

An example of how the study test array could look in each condition is shown in figure 1.

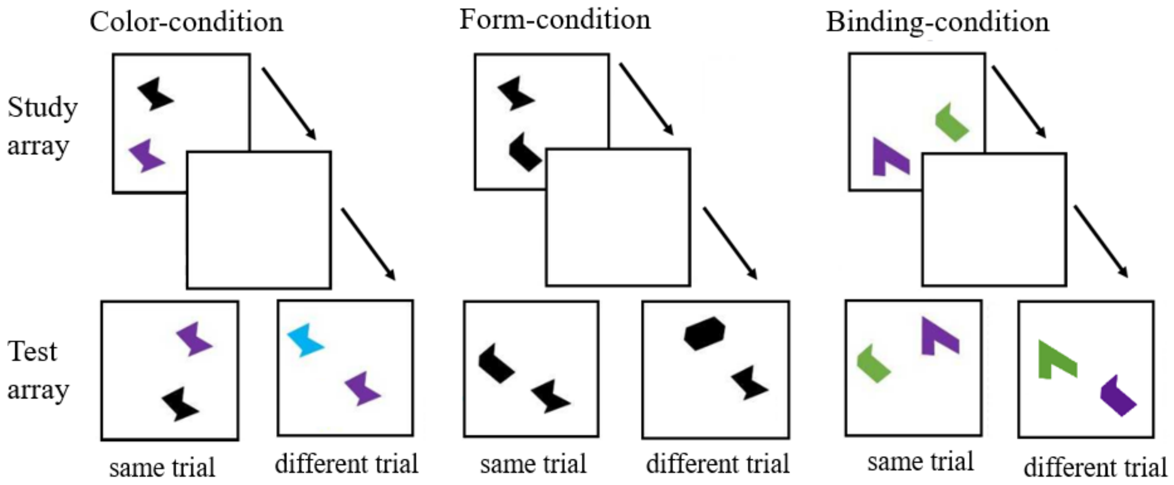

Supplementary Figure 5

Supplementary Figure 5. Study and test array in the color-, form- and binding-condition of the short-term memory binding (STMB) task. In the color-condition the colors can vary from the study to the test array, in the form-condition the forms can vary and finally, in the binding-condition forms can have different colors.

Results of the stepwise-regression to determine appropriate predictors

A forward stepwise linear regression was used to identify possible predictors of the outcome CPI out of the following candidate variables: age, GDS score, FNAT recall, FNAT trial 1, FNAT proactive interference as well as group. At each step, variables were added based on p-values.

The stepwise regression returned group, age, GDS score and FNAT recall performance as suitable predictors for CPI level.

Supplementary Table 5.

	B	SE of B	β	p
Constant	3.354	.318		<.001***
Group (HS)	.557	.145	.369	<.001***
Age	-.011	.004	-.021	.010*
GDS	.049	.017	.272	.004**
FNAT recall	-.079	.019	-.327	<.001***

Supplementary Table 5. Results of stepwise-regression for CPI to determine appropriate predictors. CPI: Complainer Profile Identification; GDS: Geriatric Depression Scale; FNAT: NOS faces-names-test recall; significance level: p<.05*, p<.01**, p<.001***
